# Emotional–behavioral difficulties and maternal psychosocial factors in preschool children with food allergy: a cross-sectional case–control study

**DOI:** 10.1007/s00431-026-06972-w

**Published:** 2026-04-29

**Authors:** Pelin Celik, Sule Buyuk Yaytokgil, Meltem Bayram Sen, Ersoy Civelek

**Affiliations:** 1 Division of Developmental and Behavioral Pediatrics, Department of Pediatrics, Ankara Yildirim Beyazit University, Bilkent City Hospital, Ankara, Turkey; 2 Division of Pediatric Allergy and Immunology, Department of Pediatrics, Ankara Research and Training Hospital, Ankara, Turkey; 3 Division of Developmental and Behavioral Pediatrics, Department of Pediatrics, Etlik City Hospital, Ankara, Turkey; 4 Division of Pediatric Allergy and Immunology, Department of Pediatrics, Memorial Hospital, Ankara, Turkey

**Keywords:** Behavioral problems, Children, Food allergy, Mother, Vulnerability

## Abstract

Food allergy (FA) is a common chronic condition, yet its behavioral and psychosocial impact in early childhood remains insufficiently understood. This cross-sectional study assessed emotional-behavioral outcomes, maternal anxiety, and perceived child vulnerability (PCV) in preschoolers with FA versus healthy controls, with subgroup analyses for food-related anaphylaxis and respiratory allergic comorbidities. A total of 186 children aged 1.5–5 years (84 with FA and 102 age- and sex-matched controls) and their mothers were included. Behavioral functioning was assessed using the Child Behavior Checklist, while maternal anxiety and PCV were measured with the State-Trait Anxiety Inventory-Trait and Child Vulnerability Scale. Children with FA demonstrated higher levels of emotional reactivity, somatic complaints, and sleep problems compared with healthy peers (all *p* < 0.05). Mothers of affected children reported higher PCV. Within the FA group, children with respiratory allergic comorbidity exhibited more pronounced internalizing, externalizing, and total behavior problems, whereas those with a history of anaphylaxis had lower attention problem scores (all *p* < 0.05). Maternal anxiety and PCV were positively associated with children’s internalizing, externalizing, and total behavioral difficulties (all *p* < 0.05). In multivariate analyses, FA was associated with higher PCV (OR = 4.31, 95% confidence interval: 1.81–10.27, *p* = 0.001), and this association was stronger in children with respiratory allergic comorbidity (OR = 8.34, 95% CI: 2.15–32.32, *p* < 0.01).

*Conclusion*: Preschool children with FA may be at increased risk for emotional and behavioral difficulties, accompanied by heightened PCV, particularly when respiratory comorbidities are present. Integrating developmental and behavioral monitoring and parent-focused psychosocial support within the clinical care may help improve outcomes for both children and their families.

**Table:** 

**What is Known:** •* Social and behavioral difficulties are well described in school-aged children and adolescents with food allergy, but data on preschoolers are limited.* • *Parental perceptions of child vulnerability influence behavioral outcomes, yet this has scarcely been studied in young children with food allergy.*	
**What is New:** • *Preschoolers with food allergy exhibit more emotional and behavioral difficulties, and their mothers report higher perceived vulnerability.* • *Respiratory comorbidities are linked to stronger perceived vulnerability and more pronounced emotional and behavioral symptoms.*

**Supplementary Information:**

The online version contains supplementary material available at 10.1007/s00431-026-06972-w.

## Introduction

Food allergy (FA) is a common chronic condition that has become increasingly prevalent in recent decades, posing a significant public health concern [1]. FA typically emerges in the early years of life—a sensitive period for brain and behavioral development—and has been linked to biological pathways relevant to neurodevelopment [2], making emotional and behavioral difficulties in affected young children an important area of concern.

Previous research has shown that school-aged children and adolescents with FA may experience lower self-esteem and social difficulties compared to their healthy peers [3]. They have also been reported to exhibit internalizing behaviors such as depression, anxious coping, separation anxiety, and alexithymia, as well as externalizing difficulties, including attention deficit hyperactivity disorder (ADHD) [2, 4–10]. However, most studies focus on school-aged and adolescent populations, whereas data on preschool-aged children with FA are limited.

Psychosocial factors, particularly parental perceptions and responses, play a crucial role in shaping a child’s emotional and behavioral outcomes. One important psychosocial factor is perceived child vulnerability (PCV), which reflects a mother’s belief that her child is fragile or at heightened risk [11]. In children with FA, although allergen avoidance prevents potentially life-threatening reactions, it may also increase parental stress and lead to overprotective parenting behaviors aimed at minimizing perceived environmental threats [12, 13]. Such overprotection and heightened PCV can restrict the child’s independence, peer interactions, and engagement in learning-supportive environments, potentially resulting in social, emotional, or behavioral difficulties [11, 14–17]. Despite its significance, PCV has not been adequately studied in parents of children with FA [16, 18].

Given these gaps, this study aimed to assess emotional–behavioral problems, PCV, and maternal anxiety in preschool-aged children with FA, and to examine whether these characteristics differed in subgroups defined by a history of anaphylaxis or respiratory allergic comorbidity. These aims address an important gap in current knowledge regarding the early behavioral and psychosocial impact of FA.

## Material and methods

### Study population

This cross-sectional case–control study was conducted in the Pediatric Allergy and Immunology and Developmental and Behavioral Pediatrics Divisions of Ankara Bilkent City Hospital between January 2021 and January 2023, following ethics committee approval (Approval No: E2-20–07).

**Fig. 1:**
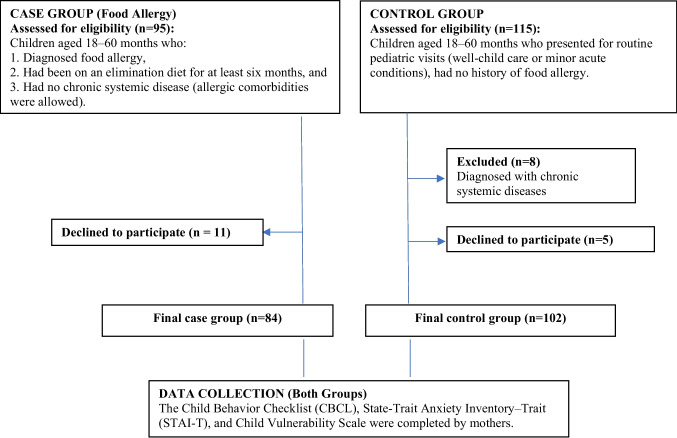
Participant flow diagram

The inclusion criteria for FA group were (1) children aged 18 to 60 months, (2) an elimination diet for at least 6 months due to FA, and (3) absence of chronic systemic diseases (comorbid allergic conditions such as atopic dermatitis or asthma were allowed). During outpatient visits, 95 eligible mother–child pairs were approached, and 84 (88.4%) agreed to participate, with non-participation mainly due to limited time or lack of interest. A total of 115 children who presented for routine pediatric visits (well-child care or minor acute conditions) were assessed for eligibility for the control group. Of these, 5 declined participation and 8 were excluded due to chronic systemic diseases. The final control group consisted of 102 mother–child pairs, with no history of FA or chronic systemic disease, and was frequency-matched to the FA group by child age and sex. The enrollment process is summarized in Fig. 1.

### Allergological workup

FA was diagnosed based on clinical history and allergological evaluations (specific IgE testing, skin prick test (SPT), and/or food challenge) following guidelines [19]. SPT was performed using commercially available solutions (ALK-Albelló, Madrid, Spain) and fresh foods via the prick-to-prick method, with wheal sizes > 3 mm considered positive [19]. Serum food-specific IgE levels were investigated using the Immulite system (Siemens Healthcare Diagnostics, Tarrytown, NY, USA), concentrations > 0.35 kU/L regarded as positive. Oral food challenges were performed according to guidelines based on the patient’s age and reaction [20, 21]. For diagnostic confirmation, an elimination diet was administered for 15–30 days, and clinical improvement was evaluated. Recurrence of symptoms upon reintroduction of the eliminated food was considered confirmation of FA. Following diagnosis, all children continued the appropriate elimination diet.

Food-induced anaphylaxis was defined according to the European Academy of Allergy and Clinical Immunology Task Force criteria [22]. Cases were classified as anaphylaxis if they showed (1) rapid onset of symptoms involving two or more organ systems after exposure to a suspected food allergen, or (2) hypotension occurring within minutes to several hours after exposure to a known food allergen. All cases were evaluated by pediatric allergists using clinical history and appropriate allergy testing, including skin prick tests, specific IgE, serum tryptase, and/or open food challenges. A diagnosis was confirmed when these assessments supported a causal link with a specific food allergen. Tryptase was measured when patients presented within 6 h of the reaction or during an open food challenge. Open food challenges were performed only in selected non-severe cases with parental consent.

Respiratory allergic comorbidity was defined as physician-diagnosed asthma and/or allergic rhinitis. Asthma was diagnosed by pediatric allergists according to Global Initiative for Asthma (GINA) criteria, based on recurrent wheezing, cough, and variable respiratory symptoms triggered by viral infections and/or allergens, and supported by the clinical response to a trial of inhaled corticosteroids [23]. Allergic rhinitis was diagnosed by pediatric allergists according to Allergic Rhinitis and its Impact on Asthma (ARIA) criteria, based on the presence of intermittent or persistent nasal symptoms, including sneezing, rhinorrhea, nasal congestion, and itching, often associated with allergen exposure [24].

### Study procedure and measures

A sociodemographic data form was used to gather comprehensive information on both the child’s and the family’s characteristics. Socioeconomic status (SES) was evaluated using the Hollingshead Redlich Index based on parental education and occupation [25]. The index score was calculated as (occupation score × 7) + (education score × 4), with higher scores indicating lower SES. For each family, the higher of the parental education and occupation levels (from either the mother or father) was used for scoring. SES scores were grouped into three categories by combining adjacent levels (high: 11–31, middle: 32–47, low: 48–77). Breastfeeding was defined as having ever received breast milk (i.e., any breastfeeding), regardless of duration or exclusivity. Household size was defined as the total number of individuals residing in the same household. Consanguinity was defined as parental relatedness, including first- or second-cousin marriage. For children with FA, additional data collected included the age at onset of FA symptoms, the age of diagnosis, the presentation of FA, the causative foods, duration of food elimination, adrenaline prescriptions, and coexisting allergic conditions.

### The Child Behavior Checklist 1^1/2^–5 (CBCL 1^1/2^–5)

The CBCL 1^1/2^–5, a 100-item parent-report instrument, was used to assess children’s emotional and behavioral problems. The instrument demonstrates 83% specificity and 66% sensitivity [26]. It has been standardized for Turkish children, with an internal consistency of 0.82 [27, 28]. The scale includes seven subscales: emotionally reactive, anxious/depressed, somatic complaints, withdrawn, sleep problems, attention problems, and aggressive behavior. The sum of scores from the emotionally reactive, anxious/depressed, somatic complaints, and withdrawn subscales constitutes the “internalizing problems score,” while the sum of attention problems and aggressive behavior scores forms the “externalizing problems score.” These seven subscale scores, along with an additional item added by the parents, make up the “total problems score.” Higher scores reflect more problematic behavior.

### Child Vulnerability Scale (CVS)

Maternal perceptions of child vulnerability and fragility were assessed using the CVS [29]. Scores ≥ 10 indicate perceived vulnerability, with higher scores reflecting greater PCV.

### State-Trait Anxiety Inventory-Trait (STAI-T)

Mothers’ anxiety levels were assessed using the STAI-T, which measures stable aspects of anxiety, like general calmness and a sense of security [30, 31]. Higher scores indicate greater anxiety, with scores exceeding 45 signifying high anxiety [32].

Following clinical evaluation in the allergy outpatient clinic, mothers who provided informed consent were invited to complete the CBCL 1^1/2^–5, CVS, and STAI-T during the same visit. The questionnaires were primarily self-administered by mothers. When needed, trained researchers provided assistance or clarification to ensure accurate and complete responses.

### Statistical analysis

Statistical analyses were performed using IBM SPSS Statistics for Windows, Version 22.0 (IBM Corp., Armonk, NY, USA). Descriptive statistics were reported as mean ± standard deviation or median (first and third quartiles) for continuous variables and as frequencies (%) for categorical variables. Normality was assessed using the Kolmogorov–Smirnov test, histograms and boxplots. Categorical variables were compared using the chi-square (*χ*^2^) test or Fisher’s exact test when appropriate. Group comparisons for continuous variables were conducted using the independent-samples *t* test or the Mann–Whitney *U* test, depending on data distribution. Spearman’s correlation was used to examine relationships between STAI-T, CVS, and CBCL scores.

Logistic regression analyses were conducted to identify factors associated with PCV (CVS scores) in the total sample and within the FA group. In the total sample, potential predictors included child age and sex (male), maternal and paternal age, parental education (< 14 years), birth order, SES (low-to-middle), STAI-T score, and FA diagnosis. In the FA group, allergy-specific variables (age at diagnosis, multiple food allergies, duration of elimination diet, history of anaphylaxis, adrenaline prescription, and respiratory allergic comorbidity) were additionally examined. Variables with *p* ≤ 0.20 in univariate analyses were entered into the multivariable model. Final models were adjusted for maternal and paternal age, parental education, SES, STAI-T score, and FA diagnosis in the total sample; and for maternal and paternal age, parental education, SES, STAI-T score, and respiratory allergic comorbidity in the FA group. Multivariable analyses were performed using backward stepwise selection. Odds ratios (ORs) and 95% confidence intervals (CIs) were reported. A *p*-value < 0.05 was considered statistically significant. Multicollinearity was assessed using variance inflation factor (VIF) and tolerance values. All VIF values were below 5 and tolerance values were above 0.2, indicating no evidence of multicollinearity.

Missing data were minimal. Analyses were performed using a complete-case approach. The number and percentage of missing values for the CBCL, STAI-T, and CVS measures are presented in Supplementary Table 1. No imputation or prorating procedures were applied.

A post hoc power analysis was performed for the CBCL total problems score based on the observed mean difference between groups. Using a two-tailed test with an alpha level of 0.05 and an effect size of 0.5, the available sample size yielded a statistical power of 92%. For CVS ≥ 10, the power analysis was conducted based on the difference between two independent group proportions. In a two-tailed analysis, the alpha error probability was set at 0.05. Considering the available sample size, the statistical power of the study was calculated as 91%.

## Results

A total of 186 mother–child pairs were enrolled (84 in the FA group and 102 controls), with no significant sociodemographic differences between groups (Table 1).

**Table 1:** Sociodemographic characteristics of the food allergy and control groups

Sociodemographic characteristics	Food allergy group *n* = 84	Control group *n* = 102	Test statistics	
			Test value	*p*
Age of child, mo, median (IQR)	28.0 (22.0–42.0)	32.5 (23.0–41.0)	*z* = − 1.039	0.30
Sex, male, *n* (%)	56 (66.7)	58 (56.9)	*χ*^2^(1) = 1.866	0.17
Gestational age, weeks, median (IQR)	39.0 (37.0–40.0)	39.0 (38.0–40.0)	*z* = − 0.970	0.33
Birth weight, g, mean ± SD	3162.10 ± 571.72	3271.21 ± 476.97	*t* (175) = − 1.384	0.17
Cesarean delivery, *n* (%)	51 (60.7)	57 (55.9)	*χ*^2^(1) = 0.397	0.53
Age of mother, years, median (IQR)	30.0 (27.3–33.0)	31.0 (28.0–33.0)	*z* = − 1.081	0.28
Age of father, years, median (IQR)	32.0 (30.0–36.0)	34.0 (31.0–37.3)	*z* = − 1.254	0.21
Mother’s education ≥ 14 years, *n* (%)	43 (51.2)	54 (52.9)	*χ*^2^(1) = 0.057	0.81
Father’s education ≥ 14 years, *n* (%)	47 (56.0)	52 (51.0)	*χ*^2^(1) = 0.457	0.50
Family structure, *n* (%)			*χ*^2^(1) = 0.019	0.89
Nuclear family	77 (91.7)	93 (91.2)		
Extended family	7 (8.3)	9 (8.8)		
Household size, median (IQR)	3.5 (3.0–5.0)	4.0 (3.0–4.0)	*z* = − 0.564	0.57
Consanguinity, *n* (%)	8 (9.5)	12 (11.8)	*χ*^2^(1) = 0.241	0.62
Birth order, median (IQR)	1.0 (1.0–2.0)	1.0 (1.0–2.0)	*z* = − 0.232	0.82
Number of siblings, median (IQR)	0.0 (0.0–1.0)	1.0 (0.0–1.0)	*z* = − 1.306	0.19
Socio-economic status (Hollingshead index), *n* (%) High Middle Low	32 (38.1) 37 (44.0) 15 (17.9)	38 (37.2) 35 (34.3) 29 (28.5)	*χ*^2^(2) = 3.564	0.17
Breastfeeding rate, *n* (%)	83 (98.8)	100 (98.0)	Fisher’s exact	1.00
Total duration of breast-feeding, mo, median, IQR	18.0 (7.5–22.0)	20.0 (17.0–24.0)	*z* = − 2.628	< 0.01
Attending kindergarten/preschool, *n* (%)	10 (11.9)	20 (19.6)	*χ*^2^(1) = 1.830	0.18

*g* gram, *IQR* interquartile range, *mo* month, *SD* standard deviation

*χ*^2^ chi-square test, *t* independent samples *t*-test, *z* Mann–Whitney *U* test, Fisher’s exact test was used when expected cell counts were < 5

Clinical characteristics of the FA group are summarized in Table 2. The median age at diagnosis was 6.0 months (IQR: 4.0–9.0), and the median duration of food elimination was 20.5 months (IQR: 14.0–29.8). Of the children, 49 (58.3%) had multiple food allergies, with hen’s egg being the most common allergen (82.1%). Additionally, 26 (31%) had a history of anaphylaxis, and 20 (23.8%) had respiratory allergic comorbidity.

**Table 2:** Clinical characteristics of children with food allergy

Clinical characteristics	Food allergy group *n* = 84
Age at onset of food allergy-related symptoms (mo), median (IQR)	4.0 (2.0–6.0)
Age at diagnosis (mo), median (IQR)	6.0 (4.0–9.0)
Multiple food allergies, n (%)	49 (58.3)
**Duration of food elimination (mo)** ^a^ Median (IQR) 6–12 months, *n* (%) 13–24 months, *n* (%) More than 24 months, *n* (%)	20.5 (14.0–29.8) 16 (19.0) 38 (45.2) 30 (35.7)
**Presentation of food allergy, *n* (%)** ^b^ Atopic dermatitis Anaphylaxis Urticaria—angioedema FPIAP FPIES	52 (61.9) 26 (31) 14 (16.7) 6 (7.1) 3 (3.6)
**Causative and eliminated foods, *n* (%)** ^c^ Hen’s egg Cow’s milk Tree nut Legumes Fruit/vegetables Other (chicken, beef, and honey)	69 (82.1) 55 (65.5) 32 (38.1) 22 (26.2) 16 (19.1) 6 (7.1)
Adrenaline prescription, *n* (%)	32 (38.1)
**Coexisting allergic conditions, *n* (%) **^d^ Atopic dermatitis Respiratory comorbidities, *n* (%) Asthma Allergic rhinitis	56 (66.7) 20 (23.8) 18 (21.4) 6 (7.1)

^a^ The longest elimination duration was taken into consideration

^b^ In 17 children, food allergy presented with a combination of multiple clinical entities

^c^ More than one food allergy may be present in the same child

^d^ Multiple allergic conditions may be present in the same child

*FPIAP* food protein–induced allergic proctocolitis, *FPIES* Food protein-induced enterocolitis syndrome, *IQR* interquartile range, *mo* months

The comparison between the FA group and the control group is presented in Table 3. Subgroup analyses within the FA group (children with versus without anaphylaxis and those with versus without respiratory allergy) are presented in Table 4.

**Table 3:** Comparison of behavioral characteristics, maternal anxiety, and perceived child vulnerability between children with food allergy and controls

Measures	Food allergy group *n*=84	Control group *n*=102	Test statistics	
			Test value	*p*
The Child Behavior Checklist, median(IQR)				
Emotionally reactive	3.50 (2.00–5.00)	2.00 (1.00–4.00)	z=−2.044	0.04
Anxious/depressed	3.00 (2.00–5.00)	2.00 (1.00–4.00)	z=−1.913	0.06
Somatic complaints	3.00 (2.00–6.00)	2.00 (1.00–4.50)	z=−2.545	0.01
Withdrawn	1.50 (0.00–3.00)	1.00 (0.00–3.00)	z=−0.182	0.86
Sleep problems	4.00 (2.00–6.00)	3.00 (1.00–5.00)	z=−2.041	0.04
Attention problems	3.00 (2.00–4.00)	3.00 (2.00–4.00)	z=−1.265	0.21
Aggressive behavior	9.00 (6.00–13.00)	7.00 (3.00–14.00)	z=−1.747	0.08
Internalizing problems	12.00 (6.25–18.75)	9.00 (4.00–17.00)	z=−1.929	0.05
Externalizing problems	13.00 (9.00–17.00)	11.00 (6.00–18.00)	z=−1.858	0.06
Total problem score	38.50 (26.25–55.00)	36.00 (18.25–53.00)	z=−1.804	0.07
Maternal State-Trait Anxiety Inventory- Trait Scale				
STAI-T, mean±SD	39.64±9.08	38.50±7.98	t(184)=0.913	0.36
STAI-T >45, n (%)	20 (23.8)	17 (16.7)	χ²(1)=1.475	0.23
The Child Vulnerability Scale				
CVS, median (IQR)	7.00 (3.00–12.00)	3.50 (1.00–7.00)	z=−3.642	<0.001
CVS ≥10, n (%)	31 (36.9)	16 (15.7)	χ²(1)=10.983	0.001

*IQR* interquartile range, CVS Child Vulnerability Scale, STAI-T State-Trait Anxiety Inventory-Trait Scale

*χ*² Chi-square test, *t* independent samples t-test, *z* Mann–Whitney U test

**Table 4:** Subgroup analyses of behavioral and parental measures according to anaphylaxis and respiratory allergy status within the food allergy group

Measures	History of anaphylaxis, yes, *n* = 26	History of anaphylaxis, no, *n* = 58	Test statistics		Respiratory allergy, yes, *n* = 20	Respiratory allergy, no, *n* = 64	Test statistics	
			Test value	*p*^1^			Test value	*p*^2^
The Child Behavior Checklist, median (IQR)								
Emotionally reactive	3.50 (1.75–6.25)	3.50 (1.75–5.00)	*z* = − 0.751	0.45	5.00 (2.25–6.00)	3.00 (1.00–5.00)	*z* = − 2.117	0.03
Anxious/depressed	3.00 (1.75–7.25)	3.00 (2.00–5.00)	*z* = − 0.395	0.69	4.50 (2.25–8.00)	3.00 (2.00–5.00)	*z* = − 2.229	0.03
Somatic complaints	4.00 (2.00–6.00)	3.00 (2.00–6.00)	*z* = − 0.953	0.34	4.00 (2.00–5.75)	3.00 (2.00–6.00)	*z* = − 0.557	0.58
Withdrawn	2.00 (0.00–3.00)	1.00 (0.00–3.00)	*z* = − 0.279	0.78	2.00 (1.00–4.75)	1.00 (0.00–2.00)	*z* = − 2.477	0.01
Sleep problems	5.00 (2.00–8.00)	3.00 (2.00–6.00)	*z* = − 1.625	0.10	5.00 (2.00–7.00)	3.00 (2.00–6.00)	*z* = − 0.991	0.32
Attention problems	2.00 (1.75–3.00)	4.00 (2.75–5.00)	*z* = − 3.076	< 0.01	4.00 (2.00–5.00)	3.00 (2.00–4.00)	*z* = − 0.912	0.36
Aggressive behavior	9.00 (5.75–14.00)	9.50 (5.75–13.00)	*z* = − 0.150	0.88	12.50 (9.00–16.75)	9.00 (5.00–13.00)	*z* = − 2.754	< 0.01
Internalizing problems	13.50 (6.00–21.25)	11.50 (6.75–16.00)	*z* = − 0.770	0.44	16.00 (10.00–22.00)	11.00 (5.25–16.00)	*z* = − 2.208	0.03
Externalizing problems	12.00 (8.75–16.25)	13.50 (8.75–18.00)	*z* = − 0.950	0.34	16.50 (12.25–20.00)	11.50 (7.00–17.00)	*z* = − 2.710	< 0.01
Total problem score	37.50 (23.75–62.75)	39.00 (27.75–54.00)	*z* = − 0.242	0.81	55.50 (35.75–65.75)	36.00 (24.00–49.75)	*z* = − 2.899	< 0.01
Maternal State-Trait Anxiety Inventory-Trait Scale								
STAI-T, mean ± SD	39.81 ± 8.60	39.57 ± 9.36	*t*(82) = − 0.111	0.91	39.35 ± 7.65	39.73 ± 9.53	*t*(82) = − 0.164	0.87
STAI-T > 45, *n* (%)	7 (26.9)	13 (22.4)	*χ*^2^(1) = 0.201	0.65	3 (15)	17 (26.6)	Fisher’s exact	0.38
The Child Vulnerability Scale								
CVS, median (IQR)	7.00 (2.00–12.00)	7.00 (3.00–13.00)	*z* = − 0.296	0.77	12.00 (6.00–13.75)	5.50 (3.00–11.75)	*z* = − 2.390	0.02
CVS ≥ 10, *n* (%)	11 (42.3)	20 (34.5)	*χ*^2^(1) = 0.472	0.49	13 (65.0)	18 (28.1)	*χ*^2^(1) = 8.898	< 0.01

*p*^1^: FA with history of anaphylaxis vs. FA without history of anaphylaxis

*p*^2^: FA with respiratory allergy vs. FA without respiratory allergy

*IQR* interquartile range, *CVS *Child Vulnerability Scale,* STAI-T* State-Trait Anxiety Inventory-Trait Scale

*χ*^2^ chi-square test, *t* independent samples *t*-test, *z* Mann–Whitney *U* test, Fisher’s exact test was used when expected cell counts were < 5

### Emotional and behavioral characteristics of children

On the CBCL, children with FA had significantly higher median scores than controls in the emotionally reactive (*p* = 0.04), somatic complaints (*p* = 0.01), and sleep problems (*p* = 0.04) subscales (Table 3). They also showed higher scores on the anxious/depressed (*p* = 0.06) and internalizing (*p* = 0.05) subscales; although these differences did not reach statistical significance.

In subgroup analyses, CBCL scores were similar between children with and without a history of food-related anaphylaxis, except for significantly lower attention problems in the anaphylaxis group (*p* < 0.01) (Table 4). In contrast, children with respiratory allergic comorbidity showed significantly higher scores in the emotionally reactive (*p* = 0.03), anxious/depressed (*p* = 0.03), withdrawn (*p* = 0.01), aggressive (*p* < 0.01), internalizing (*p* = 0.03), externalizing (*p* < 0.01), and total problems scores (*p* < 0.01) compared with those without this comorbidity.

Among children with FA, higher maternal STAI-T and CVS scores were positively associated with internalizing (*r* = 0.263, *p* = 0.02; *r* = 0.525, *p* < 0.001), externalizing (*r* = 0.245, *p* = 0.03; *r* = 0.465, *p* < 0.001), and total problems scores (*r* = 0.290, *p* < 0.01; *r* = 0.598, *p* < 0.001).

### Maternal anxiety and perceived child vulnerability

There were no significant differences in maternal STAI-T scores between the FA and control groups (Table 3). Although a higher proportion of mothers in the FA group met the cutoff for maternal anxiety, this difference was not statistically significant.

Children with FA had significantly higher median CVS scores than controls (*p* < 0.001), and a greater proportion were classified as vulnerable (*p* = 0.001). Among children with FA, those perceived as vulnerable were less likely to attend kindergarten or preschool (18.9% vs. 0%, *p* = 0.01). Median CVS scores did not differ between children with and without a history of anaphylaxis (*p* = 0.77), whereas children with respiratory allergic disease had significantly higher scores (*p* = 0.02) (Table 4).

The logistic regression analyses conducted to identify factors associated with CVS scores in the total sample and the FA group are presented in Table 5. In the overall sample, FA diagnosis was an independent predictor of higher CVS scores (OR = 4.31, 95% CI:1.81–10.27, *p* = 0.001). Within the FA group, lower maternal age, maternal education less than 14 years, and higher maternal STAI-T scores were significant predictors of increased CVS scores (*p* < 0.01, *p* = 0.01, and *p* < 0.01, respectively). Notably, respiratory allergic comorbidity was strongly associated with higher CVS scores, with an odds ratio of 8.34 (95% CI:2.15–32.32, *p* < 0.01).

**Table 5:** Univariate and multivariate analyses of variables influencing child vulnerability scale scores (< 10 vs. ≥ 10) in the total sample and the FA group

	Total sample, *n* = 186				Food allergy group, *n* = 84			
	Univariate		Multivariate*		Univariate		Multivariate**	
Risk factors	OR (95.0% C.I.)	*p*	OR (95.0% C.I.)	*p*	OR (95.0% C.I.)	*p*	OR (95.0% C.I.)	*p*
Age of child	1.00 (0.97–1.03)	0.90			0.99 (0.96–1.03)	0.70		
Sex, male	1.16 (0.58–2.29)	0.68			1.08 (0.42–2.78)	0.87		
Age of mother	0.89 (0.82–0.96)	< 0.01	0.90 (0.82–0.98)	0.02	0.84 (0.74–0.95)	< 0.01	0.82 (0.71–0.93)	< 0.01
Age of father	0.96 (0.91–1.02)	0.19			0.92 (0.84–1.10)	0.08		
Mother’s education < 14 years	2.71 (1.36–5.40)	< 0.01			3.47 (1.36–8.83)	< 0.01	4.54 (1.37–15.02)	0.01
Father’s education < 14 years	3.26 (1.62–6.57)	0.001	4.98 (2.01–12.34)	0.001	3.08 (1.23–7.72)	0.02		
Birth order	1.12 (0.74–1.71)	0.59			0.89 (0.48–1.64)	0.70		
Low or middle socio-economic status	2.44 (1.15–5.20)	0.02			2.38 (0.90–6.27)	0.08		
Maternal State-Trait Anxiety Inventory-Trait Score	1.09 (1.04–1.14)	< 0.001	1.11 (1.05–1.17)	< 0.001	1.05 (1.00–1.11)	0.06	1.09 (1.03–1.17)	< 0.01
Diagnosis of food allergy^a^	3.14 (1.57–6.29)	0.001	4.31 (1.81–10.27)	0.001				
Age at diagnosis of food allergy^b^					1.03 (0.97–1.09)	0.33		
Multiple food allergies^b^					1.21 (0.49–3.00)	0.67		
Duration of food elimination^b^					0.99 (0.95–1.03)	0.58		
History of anaphylaxis^b^					1.39 (0.54–3.60)	0.49		
Adrenaline prescription^b^					1.29 (0.52–3.20)	0.58		
Respiratory allergic comorbidity^b^					4.75 (1.63–13.81)	< 0.01	8.34 (2.15–32.32)	< 0.01

*CI* confidence interval, *OR* odds ratio

ᵃVariable assessed only in the total sample

ᵇVariable assessed only in the food allergy group

^*^Omnibus test of model coefficients was statistically significant (*χ*^2^(4) = 50.106, *p* < 0.001). − 2 log likelihood = 159.590. The model explained 23.7%–35.0% of the variance (Cox & Snell *R*^2^ = 0.237; Nagelkerke *R*^2^ = 0.350). Hosmer–Lemeshow test indicated acceptable fit (*χ*^2^(8) = 12.156, *p* = 0.144)

^**^Omnibus test of model coefficients was statistically significant (χ^2^(4) = 33.695, *p* < 0.001). − 2 log likelihood = 76.924. The model explained 33.0%–45.1% of the variance (Cox & Snell *R*^2^ = 0.330; Nagelkerke *R*^2^ = 0.451). Hosmer–Lemeshow test indicated adequate fit (*χ*^2^(8) = 10.230, *p* = 0.249)

## Discussion

To our knowledge, this is the first study to concurrently investigate emotional**–**behavioral problems and PCV in preschool-aged children with FA. In our sample, affected children exhibited higher emotional reactivity, somatic complaints, and sleep problems compared with healthy peers, indicating that emotional and behavioral difficulties emerge as early as the preschool period. Mothers were also more likely to perceive their children as vulnerable, particularly when respiratory allergic comorbidities were present. Notably, both elevated PCV and higher maternal anxiety were strongly associated with greater internalizing, externalizing, and total behavioral problems, underscoring the importance of considering parental factors when evaluating behavioral outcomes in young children with FA.

The existing literature on emotional–behavioral characteristics in children with FA predominantly focuses on school-aged children, adolescents or broad pediatric cohorts. Large cohort studies consistently report higher levels of generalized and separation anxiety, depressive symptoms, and broader internalizing difficulties among individuals with FA [4–7, 9, 10]. Likewise, a nationwide cohort of more than 600,000 individuals demonstrated increased risks of anxiety, depression, sleep problems, and eating disorders in this population [33]. More recently, a large population-based matched retrospective cohort study utilizing longitudinal electronic health records across a broad pediatric age range (0–18 years) further confirmed elevated risks of anxiety, depression, and eating disorders in this population compared with healthy controls [34]. Despite this growing body of evidence from mixed-age pediatric samples, to our knowledge, only one study to date has specifically examined emotional and behavioral outcomes in younger children. In 18-month-old infants with FA, Meldrum et al. found that non-IgE-mediated FA was associated with higher internalizing problems, with a trend toward lower social–emotional scores in IgE-mediated FA [35]. Our study extends this work by focusing on preschool-aged children (18–60 months). Consistent with emerging evidence from early childhood samples and broader pediatric cohorts, we observed higher emotional reactivity and somatic complaints—both indicators of internalizing behavior—as well as significantly more sleep problems compared with healthy controls. Although anxious/depressed and overall internalizing problem scores were higher in the FA group, these differences did not reach statistical significance, suggesting a possible but not definitive tendency toward emerging internalizing difficulties. In our sample, 62% of children with FA had atopic dermatitis, a condition frequently linked to sleep disturbances in early childhood; therefore, this comorbidity likely contributed to the higher sleep problem scores observed in this group [36]. Moreover, given the young age of our sample, elevations in emotional reactivity, somatic complaints, and sleep problems may reflect early regulatory disturbances that signal emerging internalizing susceptibility. In line with longitudinal evidence linking FA to escalating risks of generalized anxiety and depressive symptoms over time [5], and with Nemet et al.’s findings of a steadily increasing likelihood of psychological disorders in this population [33], these early manifestations may mark the onset of internalizing trajectories that become more pronounced with age. Taken together, our findings suggest that emotional–behavioral difficulties in children with FA may begin as early as the preschool years, initially presenting through somatic, emotional, and sleep-related features. This underscores the importance of careful monitoring and anticipatory guidance during early childhood. Nevertheless, these observations should be interpreted in the context of our cross-sectional design. Longitudinal studies beginning in early childhood, when FA typically emerges, are needed to validate and further clarify these developmental trajectories.

In our study, mothers of children with FA perceived their children as more vulnerable than mothers of controls, and multivariate regression analysis showed that FA increased the likelihood of higher PCV by more than fourfold. Although PCV has not been previously examined in children with FA, the rate observed in our sample (36.9%) is similar to that reported in other pediatric chronic conditions [17, 37]. Within the FA group, younger maternal age and lower maternal education levels were associated with higher PCV, suggesting that differences in experience or knowledge may shape maternal perceptions. As expected, higher maternal anxiety scores were associated with increased PCV, highlighting the role of parental psychological factors in the perception of child vulnerability. Children whose mothers perceived them as more vulnerable exhibited higher internalizing, externalizing, and total problems, consistent with prior research showing that heightened parental vulnerability perceptions relate to poorer socioemotional adjustment, depressive symptoms, anxiety, and behavioral difficulties in children with a range of medical conditions [38–41]. These findings underscore the potential impact of maternal perceptions not only on parenting behaviors but also on the child’s emotional and behavioral functioning. Notably, we found no association between elimination diet duration and PCV. Therefore, from diagnosis onward, healthcare professionals should support families in adopting a balanced approach: while ensuring safety through allergen avoidance, careful label reading, and prompt recognition of allergy-related symptoms, they should also consider the potential behavioral impact of elevated PCV. In clinical practice, identifying elevated PCV begins with explicitly exploring parental worries and assessing how FA-related concerns influence family’s daily routines and participation. Indicators of heightened PCV in parents may include disproportionate fear of accidental exposure or anaphylaxis; marked parental anxiety or overprotective behaviors despite normal physical examination findings, appropriate growth parameters, and age-appropriate developmental milestones; beliefs that the child is particularly fragile or frequently ill; excessive reassurance-seeking; frequent unscheduled healthcare visits; or restriction of the child’s participation in age-appropriate play, peer interaction, or preschool attendance to prevent allergen exposure. The potential behavioural impact of elevated PCV may be observed at the child level and can include increased emotional reactivity, anxiety symptoms, sleep disturbances, unexplained somatic complaints, separation difficulties, or delayed autonomy relative to developmental expectations. When such child-level concerns are present, brief validated behavioural screening tools (e.g., CBCL or Strengths and Difficulties Questionnaire) may assist in identifying those at risk. Once elevated PCV is identified, clinicians should acknowledge parental concerns and provide individualized, evidence-based information regarding the child’s actual medical risk and management. Practical steps may include reinforcing appropriate allergen avoidance, providing a written emergency action plan, and—when indicated—educating caregivers on correct epinephrine auto-injector use. To address potential behavioural consequences, clinicians may guide parents in maintaining consistent routines, modeling calm responses to allergy-related situations, supporting developmentally appropriate autonomy, and facilitating gradual participation in safe social activities. Referral to mental health services should be considered if emotional or behavioural symptoms persist or interfere with functioning.

It is important to determine whether FA severity or a history of anaphylaxis confers additional emotional–behavioral or psychosocial risk in children; however, existing findings in the literature remain inconsistent. Emeksiz et al. compared 30 children aged 12–62 months with food-induced anaphylaxis to healthy controls and reported higher total Aberrant Behavior Checklist scores, including increased stereotypic behaviors and hyperactivity [42]. In adolescents, Polloni et al. similarly found elevated internalizing and externalizing problems among those with anaphylaxis, whereas access to an adrenaline autoinjector appeared to relate to lower symptom levels, potentially reflecting the psychological benefit of preparedness [7]. At a population level, Nemet et al. reported that individuals with FA and a history of anaphylaxis—across childhood, adolescence, and adulthood—had a 22.5% higher cumulative risk of developing psychological disorders over time, including anxiety, depression, post-traumatic stress disorder, and sleep or eating disturbances [33]. Conversely, Karim et al.’s large twin cohort found that neither severe FA nor adrenaline prescription predicted later anxiety or depression, with most associations attributable to shared familial factors rather than allergy severity [43]. Lee et al. likewise observed no significant behavioral differences in young children with severe FA, with relative risk estimates for bullying, attention problems, and anxiety remaining below 1.0, suggesting a non-significant trend toward fewer symptoms [44]. Consistent with these findings, our data also showed no significant differences in internalizing or externalizing behaviors among preschoolers, and children with a history of anaphylaxis demonstrated slightly better attention scores. One potential explanation is that heightened parental vigilance and structured safety routines adopted following an anaphylactic reaction may create an environment that supports attentional regulation [7, 45]. These results should, however, be interpreted cautiously given the small number of children with anaphylaxis in our clinically homogeneous FA cohort. Nonetheless, examining this subgroup within a well-characterized sample provides valuable preliminary insight into early behavioral patterns following anaphylaxis—an area that remains understudied.

We also examined whether respiratory allergic comorbidities contributed to additional variation in emotional and behavioral outcomes among children with FA. In our sample, children with FA and respiratory allergy—most commonly asthma—showed significantly higher externalizing scores, including aggression, as well as elevated internalizing symptoms such as emotional reactivity, anxiety/depression, and withdrawal. These findings align with prior research indicating that asthma and allergic rhinitis are associated with increased internalizing and externalizing difficulties in early childhood. For example, Nanda et al. reported that allergic rhinitis and persistent wheezing predicted later anxiety and depressive symptoms, with greater risk when multiple allergic diseases coexisted [46]. Similarly, Jiang et al. showed that FA accompanied by allergic rhinitis or asthma was associated with a stepwise increase in ADHD risk compared with FA alone, reflecting the additional neurobehavioral burden of respiratory comorbidities [8]. Multivariate analyses in our study further showed that respiratory comorbidities were strongly associated with higher PCV, whereas no such association was found for food-related anaphylaxis. Several factors may help explain why respiratory allergies, rather than anaphylaxis, were more strongly associated with behavioral outcomes and elevated PCV. Respiratory symptoms occur more frequently, are more visible to families, and require ongoing daily management, creating repeated reminders of the child’s illness and sustaining daily parental vigilance. Moreover, respiratory allergies—particularly asthma—can disrupt daily functioning and quality of life [47], potentially heightening parental anxiety and contributing to greater PCV. In contrast, although anaphylaxis is potentially life-threatening, none of the children in our sample had experienced severe or refractory reactions, which may have led families to appraise these episodes as less threatening. Additionally, as noted by Polloni et al. [7], universal access to an adrenaline autoinjector in this subgroup may have enhanced preparedness and sense of control and reduced illness-related uncertainty, thereby contributing to lower PCV. Nevertheless, further research with larger samples is needed to confirm and extend these observations.

Previous research has reported elevated anxiety among parents of children with food-induced anaphylaxis [10]. In contrast, we did not observe differences in maternal trait anxiety between mothers of children with or without FA, nor between those with or without a history of anaphylaxis. One possible explanation relates to the nature of the anxiety measured. Our study we assessed general (trait) anxiety, which may not fully capture food allergy–specific anxiety. Generic anxiety measures, such as the STAI, have been shown to underestimate food allergy–related anxiety and may fail to identify a substantial proportion of parents reporting high condition-specific worry [48]. Therefore, the absence of group differences in trait anxiety does not preclude the presence of FA-specific anxiety. Future studies incorporating food allergy–specific instruments, such as the Worry About Food Allergy (WAFA) scales—including versions adapted for parents of preschool children—may provide a more sensitive evaluation of anxiety within the FA population. Child age may also have influenced our findings. Parents of preschool-aged children typically retain greater control over the child’s environment, and children at this stage are not yet exposed to school-related stressors such as bullying, exclusion, or absenteeism. In addition, contextual characteristics of the Turkish healthcare system—including easy access to emergency services, free healthcare provision, reliable availability of adrenaline autoinjectors, and access to allergen-safe foods—may reduce uncertainty and perceived risk, thereby buffering parental anxiety. Taken together, the measurement approach, child age, and healthcare context may help explain the absence of detectable differences in maternal trait anxiety in our sample.

Our study has several strengths. First, we used validated instruments to assess children’s emotional–behavioral difficulties and to measure maternal anxiety and PCV, enhancing the reliability of our findings. Second, the inclusion of a matched healthy control group allowed for meaningful comparisons. Third, examining subgroups of children with a history of anaphylaxis and respiratory allergies provided a more nuanced understanding of variability within the FA population. Finally, to our knowledge, this is the first study to evaluate both emotional and behavioral outcomes and PCV in preschool-aged children with FA using a matched control design, offering novel insights into early childhood outcomes in this group.

Despite these strengths, several limitations should be considered. First, all behavioral outcomes were based solely on maternal report, which may be subject to reporting bias. Second, as this was a single-center study, the generalizability of the findings to other settings and healthcare systems may be limited. Third, the cross-sectional design precludes examination of longitudinal relationships among emotional–behavioral problems, PCV, and FA; future prospective studies are needed to clarify potential causal pathways. Fourth, although the subgroup analyses yielded valuable insights, the relatively small numbers in the anaphylaxis (*n* = 26) and respiratory allergy (*n* = 20) groups may have limited statistical power, underscoring the need for replication in larger cohorts. Finally, data were collected between January 2021 and January 2023, a period that encompassed varying phases of the COVID-19 pandemic in Türkiye, including stricter public health restrictions in early 2021 and gradual normalization thereafter. Although pandemic-related restrictions or specific pandemic waves were not included as separate variables in the statistical models, both the FA and control groups were recruited during the same period and were therefore similarly exposed to these contextual factors. The two groups did not differ significantly in sociodemographic characteristics, kindergarten/preschool attendance, or maternal trait anxiety levels. These similarities indicate that both groups were exposed to comparable environmental and psychosocial conditions during the study period, reducing the likelihood that contextual factors—such as pandemic-related stressors—accounted for the observed differences. Nevertheless, the pandemic context may have influenced parental perceptions and children’s behavior and development, and this should be considered when interpreting the findings.

## Conclusion

This study provides important evidence that preschool-aged children with FA may be at increased risk for emotional and behavioral difficulties. These challenges often present through somatic complaints, emotional symptoms, and sleep-related problems, underscoring the need for early recognition. PCV was also elevated among mothers of children with FA and demonstrated meaningful associations with children’s emotional and behavioral outcomes. Notably, mothers of children with accompanying respiratory comorbidities reported particularly high PCV, alongside greater internalizing and externalizing difficulties in their children. These patterns underscore the importance of incorporating developmental**–**behavioral monitoring and anticipatory guidance for parents into routine FA management during the preschool years, with the aim of optimizing child well-being and supporting family functioning.

## Supplementary Information

Below is the link to the electronic supplementary material.

ESM 1 (DOCX 14.5 KB)

## Data Availability

The datasets generated and analyzed during the current study are not publicly available, but are available from the corresponding author on reasonable request.

## Abbreviations

ADHD: Attention deficit hyperactivity disorder
CBCL: Child Behavior Checklist
CVS: Child Vulnerability Scale
FA: Food allergy
PCV: Perceived child vulnerability
SES: Socioeconomic status
SPT: Skin prick test
STAI-T: State-Trait Anxiety Inventory-Trait
