# Supplementary material for: Emotional–behavioral difficulties and maternal psychosocial factors in preschool children with food allergy: a cross-sectional case–control study
Source: Eur J Pediatr. 2026 Apr 29;185(5):321. doi: 10.1007/s00431-026-06972-w (PMC13128720; doi:10.1007/s00431-026-06972-w)
Supplement: Supplementary file 1 — (DOCX 14.5 KB) [file 431_2026_6972_MOESM1_ESM.docx]

**Supplementary Table 1. Missing Data Counts and Percentages**

| **Variables** | **Missing** | |
| --- | --- | --- |
|  | **Count** | **Percent** |
| **The Child Behavior Checklist** S**ubscales** |  |  |
| Emotionally reactive | 1 | 0.5 |
| Anxious/depressed | 1 | 0.5 |
| Somatic complaints | 1 | 0.5 |
| Withdrawn | 2 | 1 |
| Sleep problems | 1 | 0.5 |
| Attention problems | 1 | 0.5 |
| Aggressive behavior | 1 | 0.5 |
| Total problems score | 2 | 1 |
| Externalizing Problems | 1 | 0.5 |
| Internalizing problems | 2 | 1 |
| **Maternal Anxiety Scores** | 0 | 0 |
| **Maternal Perception of Child Vulnerability Scores** | 0 | 0 |
